# SGIP1α, but Not SGIP1, is an Ortholog of FCHo Proteins and Functions as an Endocytic Regulator

**DOI:** 10.3389/fcell.2021.801420

**Published:** 2021-12-24

**Authors:** Sang-Eun Lee, Eunji Cho, Soomin Jeong, Yejij Song, Seokjo Kang, Sunghoe Chang

**Affiliations:** ^1^ Department of Physiology and Biomedical Sciences, Seoul National University College of Medicine, Seoul, South Korea; ^2^ Neuroscience Research Institute, Seoul National University College of Medicine, Seoul, South Korea; ^3^ Department of Biochemistry and Biomedical Sciences, Seoul National University College of Medicine, Seoul, South Korea

**Keywords:** clathrin-mediated endocysotis (CME), membrane tubulation, FCHo proteins, SGIP1, SGIP1α

## Abstract

Src homology 3-domain growth factor receptor-bound 2-like interacting protein 1 (SGIP1), originally known as a regulator of energy homeostasis, was later found to be an ortholog of Fer/Cip4 homology domain-only (FCHo) proteins and to function during endocytosis. SGIP1α is a longer splicing variant in mouse brains that contains additional regions in the membrane phospholipid-binding domain (MP) and C-terminal region, but functional consequences with or without additional regions between SGIP1 and SGIP1α remain elusive. Moreover, many previous studies have either inadvertently used SGIP1 instead of SGIP1α or used the different isoforms with or without additional regions indiscriminately, resulting in further confusion. Here, we report that the additional region in the MP is essential for SGIP1α to deform membrane into tubules and for homo-oligomerization, and SGIP1, which lacks this region, fails to perform these functions. Moreover, only SGIP1α rescued endocytic defects caused by FCHo knock-down. Thus, our results indicate that SGIP1α, but not SGIP1, is the functional ortholog of FCHos, and SGIP1 and SGIP1α are not functionally redundant. These findings suggest that caution should be taken in interpreting the role of SGIP1 in endocytosis.

## Introduction and Results

**FIGURE 1:**
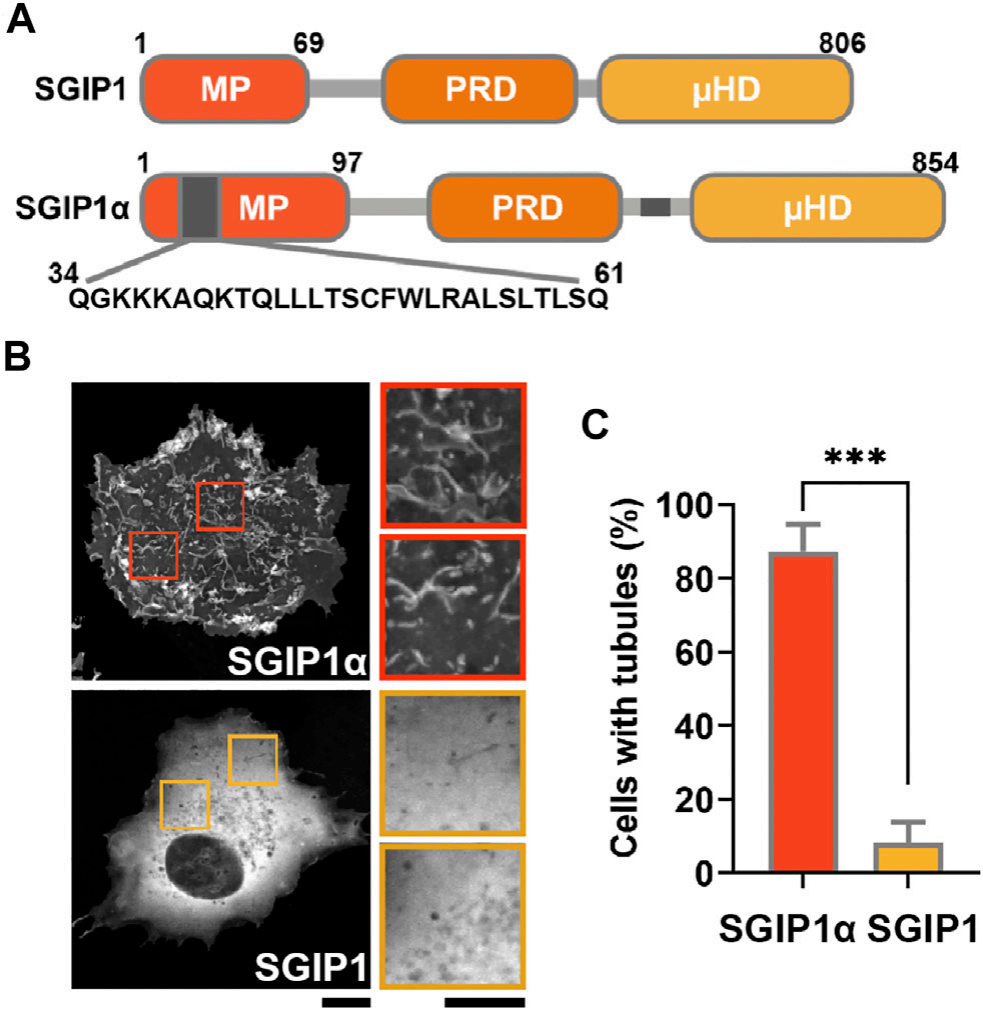
SGIP1α, but not SGIP1 deforms membrane into narrow tubules. **(A)** Schematic diagram of the domain structure of SGIP1 and SGIP1α constructs. Gray regions indicate the additional regions in the MP domain (a.a. 34–61) and the C terminal domain (a.a. 550–569). **(B)** Representative images of EGFP-tagged SGIP1 and SGIP1α expressed in COS7 cells, imaged 48 h after transfection. Scale bar: 10 and 2 μm, respectively. **(C)** Quantification of the percentage of cells with tubules in **(B)**. EGFP-tagged SGIP1α but not EGFP-SGIP1 expressed in COS7 cells deformed membrane into tubules. Cells were counted as tubule-positive when there were more than 5 visually-confirmed tubules, each ≥1.5 μm in length. Error bars indicate mean ± s.e.m. *n* = 3 (50 cells per construct for each independent experiment). ***p < 0.001, Student’s *t*-test.

SGIP1 is specifically expressed in the brain and is known to function in energy homeostasis (Trevaskis et al., 2005). It was discovered in the hypothalamus of lean and obese *Psammomys obesus* (the Israel sand rat), and antisense against hypothalamic SGIP1 mRNA inhibited food intake and led to decreased bodyweight (Trevaskis et al., 2005). Although it is not clear how SGIP1 acts as a regulator of energy balance, increased hypothalamic SGIP1 gene expression is known to be a common physiological feature of obesity and diabetes (Trevaskis et al., 2005; Cummings et al., 2012). SGIP1 consists of an N-terminal MP domain, followed by a proline-rich domain (PRD) and a C-terminal μ-homology domain (μHD) (Figure 1A). Previous studies identified SGIP1 as an ortholog of FCHo1/2 due to the similarity of its domain structure to that of FCHo proteins and its interaction with endocytic proteins Eps15 (Fazioli et al., 1993), intersectin (Dergai et al., 2010), and AP-2 (Uezu et al., 2007; Hollopeter et al., 2014). FCHo1/2 proteins are key endocytic initiators of clathrin-mediated endocytosis (CME) and contain a membrane-tubulating extended FCH/FCH and BAR domain (EFC/F-BAR) and a C-terminal μHD, which interacts with the endocytic adaptor/scaffold, Ede1/Eps15 (Henne et al., 2010; Uezu et al., 2011). However, how SGIP1 plays in two seemingly unrelated cellular processes as regulators of energy balance as well as CME remains enigmatic.

SGIP1α was later identified as the longer splice variant of SGIP1, and compared to SGIP1, it contains two additional regions: a 28 amino acid (a.a.) sequence in the MP domain (^34^QGKKKAQKTQLLLTSCFWLRALSLTLSQ^61^) and a 20 a.a sequence in the C-terminal region (^550^ENEQPSLVWFDRGKFYLTFE^569^) (Uezu et al., 2007). Although the MP domain does not show any sequence homology with the EFC/F-BAR domain in FCHo1/2, the BAR domain in FBP17, and the ENTH domain in epsin (Stimpson et al., 2009), SGIP1α was shown to deform the plasma membrane into a narrow tubular structure *via* its MP domain (Uezu et al., 2007). SGIP1α also interacts with Eps15 (Fazioli et al., 1993), intersectin (Dergai et al., 2010), and AP-2 (Hollopeter et al., 2014), further supporting its role in CME (Uezu et al., 2007; Li et al., 2011).

Surprisingly, we have found that most previous studies regarding SGIP1 either inadvertently used SGIP1 instead of SGIP1α or used different SGIP1 isoforms indiscriminately (Supplementary Table S1). For example (854 a.a.) (Hollopeter et al., 2014; Dvorakova et al., 2021; Mishra et al., 2021) and (806 a.a) (Reider et al., 2009; Stimpson et al., 2009; Henne et al., 2010; Umasankar et al., 2014; Hájková et al., 2016; Ma et al., 2016) mouse SGIP1 isoforms were used indiscriminately in many studies, although the 806 isoform lacks the additional regions in the MP and C-terminal regions. In addition, in humans, instead of the 859 a.a. isoform that contains both additional regions listed above, an (828 a.a.) (Trevaskis et al., 2005; Dergai et al., 2010; Shimada et al., 2016; Petko et al., 2018; Zhang et al., 2018) isoform that lacks the above additional region in the MP domain was used without distinction in many studies. Despite this confusion, there have been no studies comparing SGIP1 and SGIP1α, and the functional consequences with or without additional regions are unknown. Since studies of the role of SGIP1 in CME have often yielded conflicting results (Henne et al., 2010; Hollopeter et al., 2014), we suspected that a failure to distinguish between the different splicing variants might be the cause of the discrepancies.

Currently, no specific antibody against SGIP1α is available, and our attempts to raise the antibody failed due to the lack of antigenicity of the additional regions that distinguish SGIP1α from SGIP1. To see whether SGIP1α and SGIP1 are differentially expressed, we generated pairs of primers targeting the common region of the two isoforms and the additional 28 a.a. in the MP domain of SGIP1α and performed reverse transcriptase-quantitative polymerase chain reaction (RT-qPCR). We found that SGIP1α corresponded to 37.6% of the total SGIP1 mRNA levels expressed in mouse hippocampal neurons (Supplementary Figure S1A).

Proteins containing N-terminal BAR and EFC/F-BAR domains regulate membrane trafficking events by inducing membrane tubulation (Itoh and Decamilli, 2006). Consistent with a previous report (Uezu et al., 2007), we found that EGFP-tagged mouse SGIP1α readily deformed the membrane into narrow tubules in COS7 cells.

**FIGURE 2:**
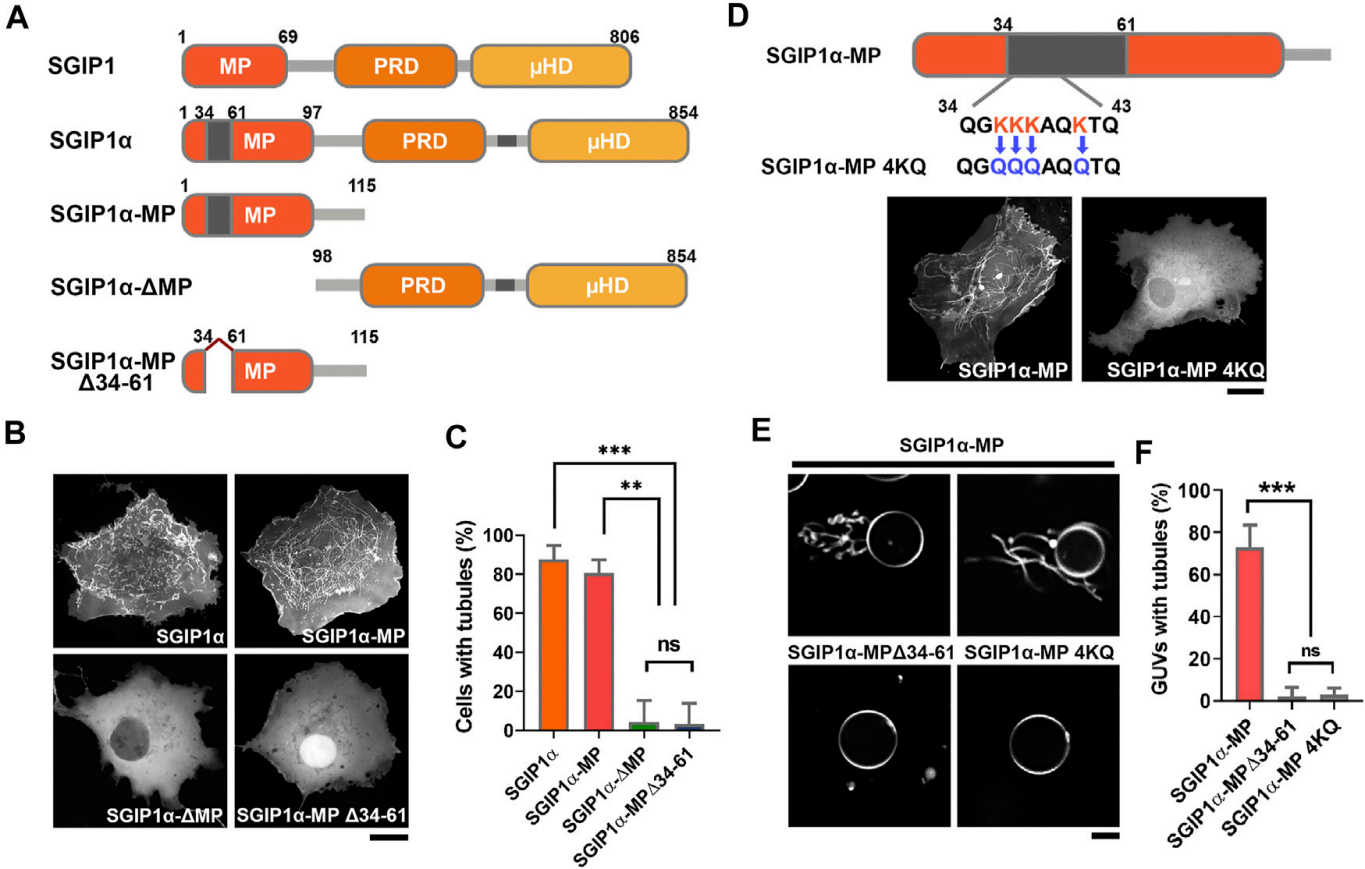
The additional 28 amino acids in the MP domain of SGIP1α are essential for membrane tubulation. **(A)** Schematic diagram of the domain structure of SGIP1, SGIP1α, SGIP1α-MP, SGIP1α-ΔMP and SGIP1α-MP Δ34-61 used in this study. **(B)** Representative images of EGFP-tagged SGIP1α, SGIP1α-MP, SGIP1α-ΔMP, and SGIP1α-MPΔ34-61 in COS7 cells. Scale bar: 10 μm. **(C)** Cells with tubules (%) of each SGIP1 construct. One-way ANOVA followed by Tukey’s HSD test. *n* = 3 (50 cells per construct for each independent experiment). (D) Schematic diagram of SGIP1α MP 4KQ construct and representative images of EGFP-tagged SGIP1α MP and 4KQ in COS7 cells. Scale bar: 10 μm. **(E)** GUV tubulation assay *in vitro*. Representative images of POPC/DOPE/DOPS/DOGS-NTA Ni/DiI GUV of purified His-tagged SGIP1α-MP, SGIP1α-MP Δ34-61, and SGIP1α-MP 4KQ. Scale bar: 10 μm. **(F)** GUV with tubules (%) of each SGIP1 construct. *n* = 3 (50 cells per construct for each independent experiment). *p < 0.05; **p < 0.01; ***p < 0.001. Error bars indicate mean ± s.e.m.

In contrast, EGFP-tagged SGIP1, SGIP1, which lacks additional regions, failed to do so, resulting in a diffuse cytoplasmic expression pattern (Figures 1B,C). These results suggest that the additional region in the MP domain is required for the membrane tubulating activity of SGIP1α. We thus transfected COS7 cells with GFP-tagged full-length mouse SGIP1α, SGIP1α-∆MP, SGIP1α-MP, and SGIP1α-MP∆34-61 (a deletion mutant lacking 28 a.a. sequence in the MP domain) and found that full-length SGIP1α and SGIP1α-MP induced membrane tubules while SGIP1α-∆MP, and SGIP1α-MP∆34-61 did not, thus confirming that the additional region in the MP is critical for forming membrane tubules (Figures 2A–C).

A charge distribution plot showed that the additional region in the MP domain is positively charged (charge at pH 7 = +4.5) and contains four closely located lysine residues, three of which are in a row (K36/37/38) (Supplementary Figure S1B). We replaced the four lysine residues with neutral glutamine (K36/37/38/41Q; 4KQ, charge at pH 7 = +0.6, Figure 2D), and found that 4KQ failed to induce membrane tubules, indicating that the positively charged residues in the MP domain are required for forming membrane tubules (Figure 2D), which was further confirmed by *in vitro* vesicle tubulation assay using the giant unilamellar vesicle (GUV) (Figures 2E,F).

Proteins containing EFC/F-BAR and BAR domains dimerize into a curved structure that either induces or stabilizes the membrane curvature (Dawson et al., 2006; Henne et al., 2007). Since the MP domain binds to membrane phospholipids and deforms membranes into tubules [(Uezu et al., 2007; Uezu et al., 2011), Figure 2B], it may homo-oligomerize. We found that HA-SGIP1α-MP interacted with GFP-SGIP1α-MP, but not with GFP or GFP-SGIP1α-MP∆34-61 (Supplementary Figure S1C). These results indicate that SGIP1α can form homo-oligomers *via* its MP domain, and that the additional region in the MP domain is essential for this.

The muniscin protein family including FCHo1/2 and Syp1 are known as key endocytic initiators of CME (Reider et al., 2009; Stimpson et al., 2009; Henne et al., 2010; Umasankar et al., 2012). Although SGIP1 was identified as an ortholog of FCHo1/2, previous attempts to test whether SGIP1 can replace the function of FCHo proteins, however, resulted in conflicting findings (Henne et al., 2010; Hollopeter et al., 2014). Since we found that the additional region in the MP domain is critical for the formation of homo-oligomers and membrane tubules (Figures 2B,C), we reasoned that the discrepancies may have arisen from the use of different splicing variants.

**FIGURE 3:**
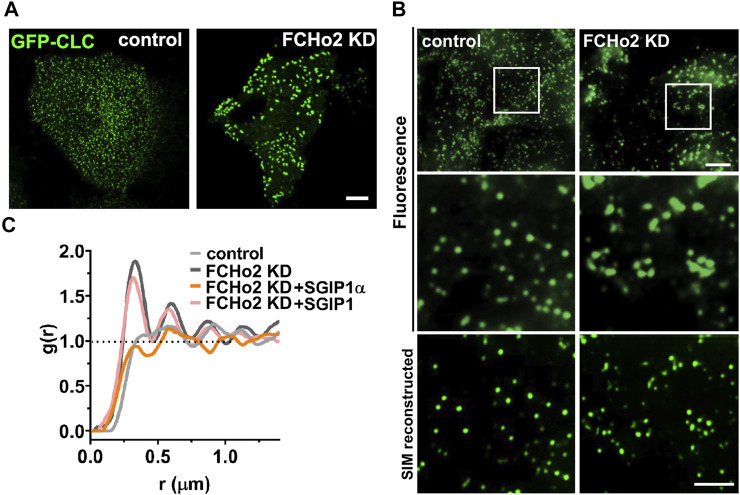
SGIP1α, not SGIP1, can reverse the altered clathrin patterning in FCHo2 KD cells. **(A)** Representative fluorescence images of EGFP-tagged clathrin light chain (CLC) in HeLa cells. HeLa cells were transfected with either control or siRNA FCHo2 and EGFP-tagged CLC. Scale bar: 10 μm. **(B)** Representative SIM reconstructed images of clathrin in control and FCHo2 KD HeLa cells. Scale bars: 5 and 2 μm, respectively. **(C)** The pair correlation function g(r) plot of control, FCHo2 KD, FCHo2 KD + SGIP1α, and FCHo2 KD + SGIP1.

To test this idea, we knocked down FCHo2 in HeLa cells (Supplementary Figure S2A; Supplementary Table S2), in which FCHo2 is predominantly expressed and FCHo1 expression is virtually undetectable (Uezu et al., 2011; Umasankar et al., 2012; Umasankar et al., 2014). In a previous study, AP2-positive puncta persisted after KD of FCHo1/2 and generally increased in size and were irregularly distributed (Umasankar et al., 2012; Umasankar et al., 2014). We confirmed that KD of FCHo2 led to enlarged and irregularly distributed clathrin puncta (Figure 3A). Structured illumination microscopy (SIM) observation further showed that enlarged puncta were indeed clustered clathrins, and that clathrins in FCHo2 KD cells formed an uneven pattern of irregular clusters compared to the random and even distribution in control cells (Figures 3B,C).

To compare the clathrin distributions quantitatively, we performed a point pattern analysis using the R statistical program. This indicated clustered patterning in FCHo2 KD in the pair correlation function g(r) whereas the patterning was regular in the control cells (Figure 3C). Then we tested whether co-expressing mouse SGIP1α or SGIP1 could rescue the distinctive clathrin patterning in FCHo2 KD cells. Only expression of SGIP1α but not SGIP1, was able to reverse the altered clathrin patterning in FCHo2 KD cells (Figure 3C and Supplementary Figure S2B), and we obtained similar results using the AP2σ subunit (Supplementary Figure S2B). These results together with the homo-oligomerization and membrane tubulation results strongly suggest that SGIP1α, but not SGIP1, is a functional ortholog of FCHo proteins, and plays a similar role to FCHo1/2 during CME.

**FIGURE 4:**
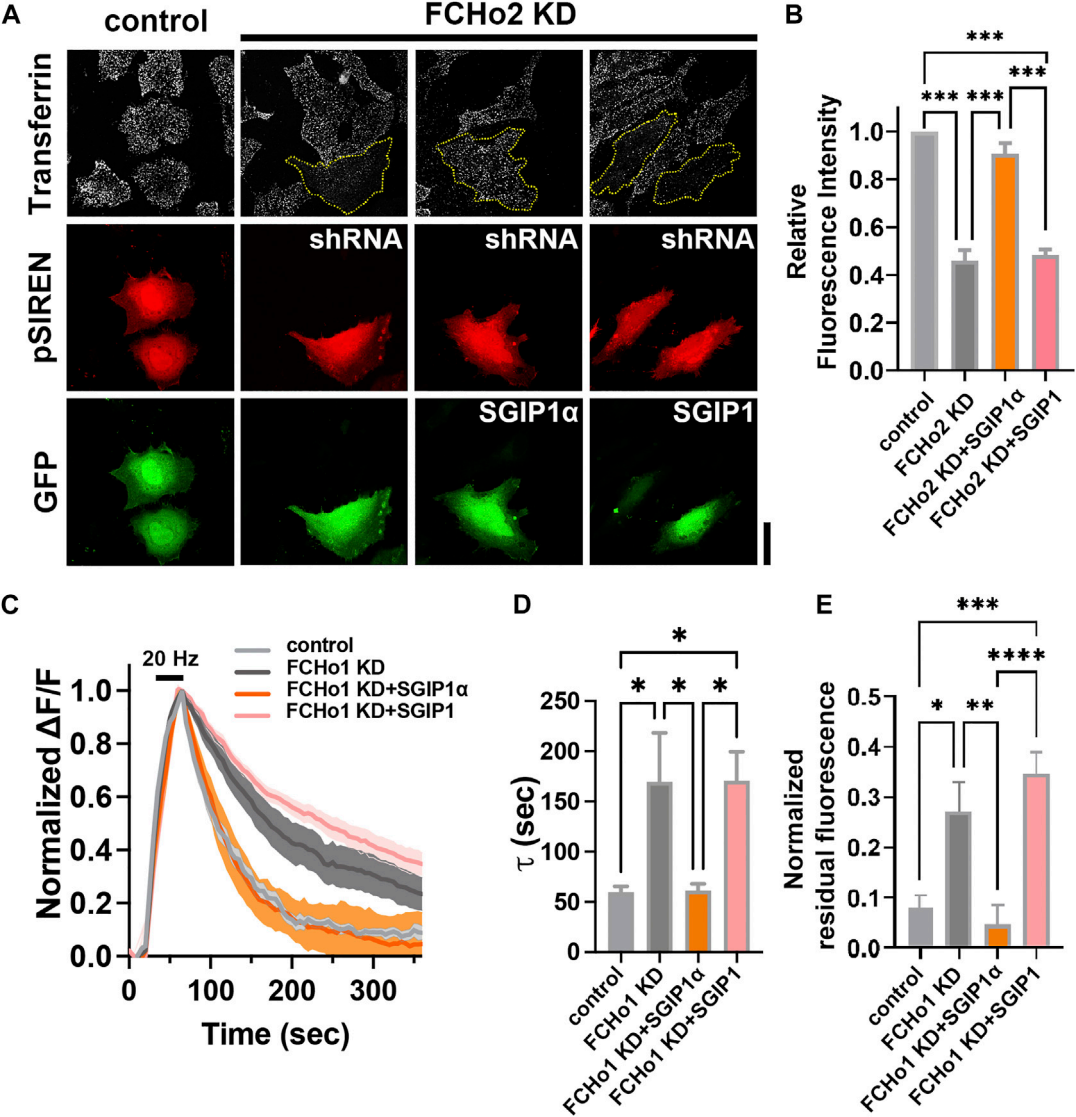
SGIP1α, not SGIP1, rescues the endocytic defects caused by FCHo depletion. **(A)** Representative fluorescence images of transferrin uptake in HeLa cells. Yellow dotted lines indicate cells transfected with shRNA-FCHo2. Scale bar: 20 μm. **(B)** A plot of relative fluorescence signals of transferrin-Alexa647 measured at 5 min of transferrin treatment in HeLa cells in **(A)**. One-way ANOVA followed by Tukey’s HSD test. *n* = 3 (15 cells per construct for each independent experiment). ***p < 0.001. Error bars indicate mean ± s.e.m. **(C)** Average synaptophysin1-pHluorin fluorescence intensity profiles in neurons expressing each construct, plotted as ΔF/F against time, after stimulation with 900 APs at 20 Hz (dark bar). Fluorescence values were normalized to the maximal fluorescence signal in each condition (control; *n* = 9 for control; *n* = 7 for FCHo1 KD; *n* = 9 for FCHo1 KD + SGIP1α; *n* = 9 for FCHo1 KD + SGIP1). **(D)** τ values for the decay of synaptophysin1-pHluorin in each experiment. τ = 59.78 ± 5.49 s for the control; τ = 169.4 ± 48.91 s for FCHo1 KD; τ = 61.29 ± 6.81 s for FCHo1 KD + SGIP1α; τ = 170.8 ± 28.63 s for FCHo1 KD + SGIP1; One-way ANOVA followed by Tukey’s HSD test. control and FCHo KD *p = 0.0307, FCHo1 KD and FCHo1 KD + SGIP1α *p = 0.0337, FCHo1 KD + SGIP1α and FCHo1 KD + SGIP1 *p = 0.0191, control and FCHo1 KD + SGIP1 *p = 0.0172. **(E)** The residual fluorescence at 360 s of endocytosis in each experiment. One-way ANOVA followed by Tukey’s HSD test. control and FCHo1 KD *p = 0.0148, FCHo1 KD and FCHo1 KD + SGIP1α **p = 0.0041, FCHo1 KD + SGIP1α and FCHo1 KD + SGIP1 ****p < 0.0001, control and FCHo1 KD + SGIP1 ***p = 0.0001.

Finally, we tested whether SGIP1α and/or SGIP1 rescued the endocytic defects caused by FCHo2 depletion (Figures 4A,B). HeLa cells were transfected with shRNA-FCHo2 and KD efficiency was confirmed by immunoblotting (Supplementary Figure S2C; Supplementary Table S2). We found that FCHo2 KD largely but not completely inhibited transferrin uptake in HeLa cells, and this was rescued by co-expression of mouse SGIP1α but not by coexpression of SGIP1 (Figures 4A,B). Coexpression of SGIP1α-4KQ or -∆34-61 also failed to rescue the endocytic defects caused by FCHo2 KD (Supplementary Figure S3). This result was further corroborated by observations of cultured rat hippocampal neurons. To quantitatively estimate endocytosis, we used synaptophysin1-pHluroin, a pH-sensitive fluorescent exo-endocytic reporter. We used shRNA instead of siRNA for sustained expression. We first attempted to express all constructs by quadruple transfection (shRNA-FCHo1 and shRNA-FCHo2 (both targeting rat isoforms)+SGIP1(α)+synaptophsyin1-pHluorin) but found it to be experimentally unreliable. Thus, we decided to knock-down FCHo1 only (Supplementary Figures S4A,B; Supplementary Table S2). Then, hippocampal neurons were co-transfected with shRNA-FCHo1, synaptophysin1-pHluorin, and either mouse SGIP1α or SGIP1 at DIV 9. Between DIV 16–18, exocytosis was evoked by 20 Hz field stimulation for 45 s, and the kinetics of endocytosis was measured (Figures 4C–E). We found that endocytosis was significantly slowed in the FCHo1 KD neurons, and co-transfection with SGIP1α but not SGIP1 rescued the defects (τ = 59.78 ± 5.49 s for the control; τ = 169.4 ± 48.91 s for FCHo1 KD; τ = 61.29 ± 6.81 s for FCHo1 KD + SGIP1α; τ = 170.8 ± 28.63 s for FCHo1 KD + SGIP1; Figures 4C,D). The residual fluorescence due to impaired endocytosis in the FCHo1 KD neurons was also only reversed by SGIP1α (Figures 4C,E). These results confirm that SGIP1α, not SGIP1, is a functional ortholog of FCHo1/2 and that SGIP1 and SGIP1α are not functionally redundant.

## Discussion

In this study, we have presented a series of experimental evidence that SGIP1 and its longer splicing variant, SGIP1α, are not functionally redundant, and that SGIP1α, not SGIP1, is a functional ortholog of FCHo proteins. We showed that an additional sequence in the MP domain of SGIP1α is crucial for conferring the functional difference between them.

After being viewed as a candidate gene for energy homeostasis and a determinant of obesity-related traits, SGIP1 was identified as an ortholog of FCHo1/2 and thus was thought to play a role in CME (Uezu et al., 2007; Hollopeter et al., 2014). Although most previous studies described SGIP1 as an endocytic regulator, there have been conflicting findings regarding its role in CME (Henne et al., 2010; Hollopeter et al., 2014). The story became even more complicated when a longer splicing variant of SGIP1, SGIP1α, was identified. Compared to SGIP1, SGIP1α contains two additional regions: 28 a.a. in the MP domain and 20 a.a. in a C-terminal region (Uezu et al., 2007).

We found that SGIP1α mRNAs corresponded to 37.6% of the total SGIP1 mRNA expressed in mouse hippocampal neurons, albeit SGIP1α appeared to be dominant at the protein level (Lee et al., 2019). We further found that only SGIP1α formed membrane tubules and the SGIP1α MP domain with the additional region formed a homo-oligomer. Moreover, only SGIP1α reversed the structural defects of clathrin/AP2σ subunit and further endocytic defects in FCHo-depleted cells. Since only SGIP1α could replace the function of FCHo in hippocampal neurons, SGIP1α, but not SGIP1, seems to play a role in endocytosis in the nervous system. Indeed, we previously reported that KD of pan-SGIP1 in hippocampal neurons caused the endocytic defects of synaptic vesicle that contains synaptotagmin-1, and it was fully rescued by co-expression of the shRNA-resistant form of SGIP1α in KD neurons (Lee et al., 2019). Since synaptotagmin-1 is known as an essential calcium sensor for neurotransmission, the role of SGIP1α for proper internalization of synaptotagmin-1 represents a pivotal role of SGIP1α in synaptic endocytosis (Lee et al., 2019).

Polybasic amino acid patches have been found in several membrane remodeling proteins. For example, the helical region 1(HR1) domain of cavin proteins contains a basic surface patch that interacts with negatively charged poly-phosphoinositides and coordinates the additional membrane-binding sites to facilitate membrane association and remodeling (Kovtun et al., 2014). In addition, endophilin, an N-BAR domain protein, is known to generate a high curvature membrane by interacting with phospholipids via its hydrophilic face consisting of several basic residues and is embedded into the bilayer via the hydrophobic patch of amphipathic stretch (Farsad et al., 2001). Similarly, it is possible that the positively charged N-terminal patch containing four closely located lysine residues in the MP domain of SGIP1α binds to phospholipids in the plasma membrane and adjacent hydrophobic regions are partially embedded in the lipid bilayer, deforming the lipid bilayer into narrow tubules.

Our current study clearly shows that the additional region in the MP domain of SGIP1α is essential for its role as a CME regulator, and hence that SGIP1, as it lacks this region, plays no role in CME and cannot replace the function of FCHo proteins, thus SGIP1α and SGIP1 are not functionally redundant. Due to the inadvertent uses of different splicing variants irrespective of the presence or absence of additional regions in previous studies, our results strongly indicate that caution should be taken when using different SGIP1 isoforms as endocytic regulators. Given that SGIP1 has been found to function in two seemingly unrelated processes; energy balance and CME, it is tempting to speculate that different isoforms of SGIP1 may function in different cellular contexts although it certainly requires further studies.

## Methods

### Cell Culture and Transfection

COS7 cells (Korean Cell Line Bank, Seoul, South Korea), HEK293T cells (ATCC, Manassas, VA), and HeLa cells (Korean Cell Line Bank) were grown in Dulbecco’s Modified Eagle’s medium (DMEM) (Invitrogen, Carlsbad, CA) supplemented with 5% fetal bovine serum at 37°C and 5% CO_2_. COS7 cells were plated on coverslips 6 h (hr) before transfection in only DMEM (Invitrogen). Cells were transfected using PEI (MW 4000, 1 mg/ml) (Polysciences, Warrington, PA) in a 1:4 ratio of total DNA (μg) to PEI (μl). For gene silencing experiments, cells were transfected with small-interfering RNA (siRNA) using siGENOME SMARTpools (Dharmacon, Lafayette, CO) against human FCHo2 (M-024508-01-0005) or with small-hairpin RNA (shRNA) targeting rat FCHo1 or human FCHo2 using PEI (See Supplementary Table S2 for si- and shRNA sequences). RNAi oligos were delivered using RNAiMAX (Invitrogen) to a final concentration of 50 nM. Cells typically received two doses of siRNA with a 1-day interval and were used up to 3 days later.

### Primary Neuron Culture and Transfection

All the animal experiments were performed according to the IACUC (Institute of Animal Care and Use Committee) guidelines of Seoul National University. Detailed methods for primary neuron culture and transfection are provided in the Supplementary Methods.

### RT-qPCR

Total RNA from rat hippocampal neurons was isolated using RNeasy TRIzol (Invitrogen). Detailed methods are provided in the Supplementary Methods.

### Sequence Charge Distribution Analysis

To analyze the charge distribution of mouse SGIP1α, a charge distribution plot was demonstrated by EMBOSS explorer, a graphical user interface to the EMBOSS suite of bioinformatics tools (http://emboss.bioinformatics.nl/cgi-bin/emboss).

### Homo-Oligomerization Assay

To test whether SGIP1α-MP can form homo-di/oligomer, we conducted a co-immunoprecipitation assay. See Supplementary Methods for details.

### Immunocytochemistry

HeLa cells plated on PDL-coated coverslips were washed in pre-warmed PBS two times and fixed in 4% paraformaldehyde containing 4% sucrose for 15–20 min at room temperature. The samples were incubated with 10% BSA for 45 min for blocking and then incubated with primary antibody for 2 h in 37°C incubator. After being rinsed in PBS, they were probed with a secondary antibody (Alexa Fluor-488 or Alexa Fluor-647) for 45 min at room temperature. The samples then were mounted on the slide classes, using mounting medium (DAKO, Carpinteria, CA).

### Cell Imaging

To detect tubules, clathrin puncta, or transferrin in SGIP1α overexpressing cells, cells were fixed and mounted as described above. Images were acquired with a 488 or 561 nm laser using a spinning disk confocal microscope (ECLIPSE Ti-E, Nikon, Tokyo, Japan) with an oil immersion objective lens (Plan Apo 60× N.A. 1.40), and a Neo sCMOS camera (Andor Technology, Belfast, Northern Ireland) at room temperature. In the case of AP2σ subunit imaging, transfected cells were transferred to an imaging chamber in pre-warmed Tyrode’s solution containing the following: 136 mM NaCl, 2.5 mM KCl, 2 mM CaCl_2_, 1.3 mM MgCl_2_, 10 mM HEPES, and 10 mM glucose, pH 7.3.

### Structured Illumination Microscopy (SIM) Imaging and Data Processing

Fixed cells were mounted on slide glasses by mounting medium (DAKO) and were imaged using an N-SIM microscope (ECLIPSE Ti-E, Nikon) equipped with an oil immersion TIRF objective lens (Apo TIRF 100× N.A. 1.49), and an EMCCD camera (iXon DU-897, Andor Technology).

### Pattern Analysis

For statistical analysis of spatial point patterns of clathrin puncta obtained from SIM imaging, we used *spatstat*, a package in R. For pair correlation function, we used g(r), the probability of observing a pair of points separated by a distance r, divided by the corresponding probability for a Poisson process.

$$\text{g}\left(r\right)=\frac{K\prime \left(r\right)}{2\pi r}$$

where value g(r) = 1 corresponds to complete randomness and values g(r) > 1 suggest clustering or attraction at distance r, while values g(r) < 1 suggest inhibition or regularity.

### Protein Purification

For protein purification, mouse SGIP1α, SGIP1, SGIP1α-MP (aa 1–115), SGIP1α-MP 4KQ, and SGIP1a-MP Δ34-61 were subcloned into the pET28a. Detailed methods are provided in the Supplementary Methods.

### GUV Preparation and Imaging

GUVs were prepared according to the gel-assisted GUV protocol (Weinberger et al., 2013). Detailed methods for GUV preparation and imaging are provided in the Supplementary Methods.

### Transferrin Uptake Assay

HeLa cells were incubated in serum-free DMEM/HEPES containing 20 mg/ml Transferrin-647 (Molecular Probe, Eugene, OR) for 15 min on ice and imaged using a spinning disk confocal microscope (Nikon). See Supplementary Methods for details.

### pHluorin Endocytosis Assay and Image Analysis

Detailed methods for pHluorin endocytosis assay and image analysis are provided in the Supplementary Methods.

## Data Availability

The original contributions presented in the study are included in the article/Supplementary Material, further inquiries can be directed to the corresponding authors.
