# Supplementary figures and images for: SGIP1α, but Not SGIP1, is an Ortholog of FCHo Proteins and Functions as an Endocytic Regulator
Source: Front Cell Dev Biol. 2021 Dec 24;9:801420. doi: 10.3389/fcell.2021.801420 (PMC8740024; doi:10.3389/fcell.2021.801420)

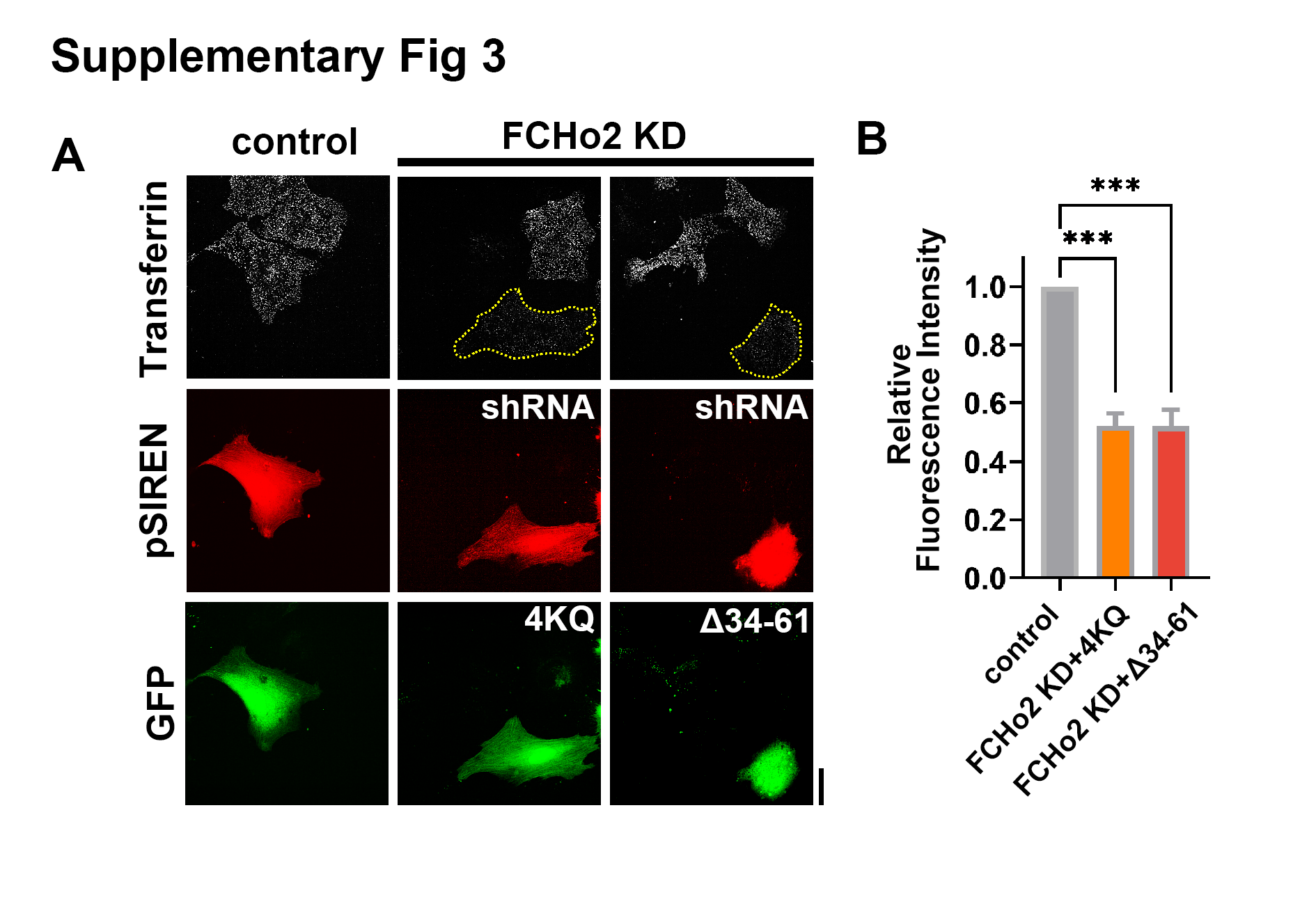

Supplement: Supplementary file 1 [file Image3.tif]

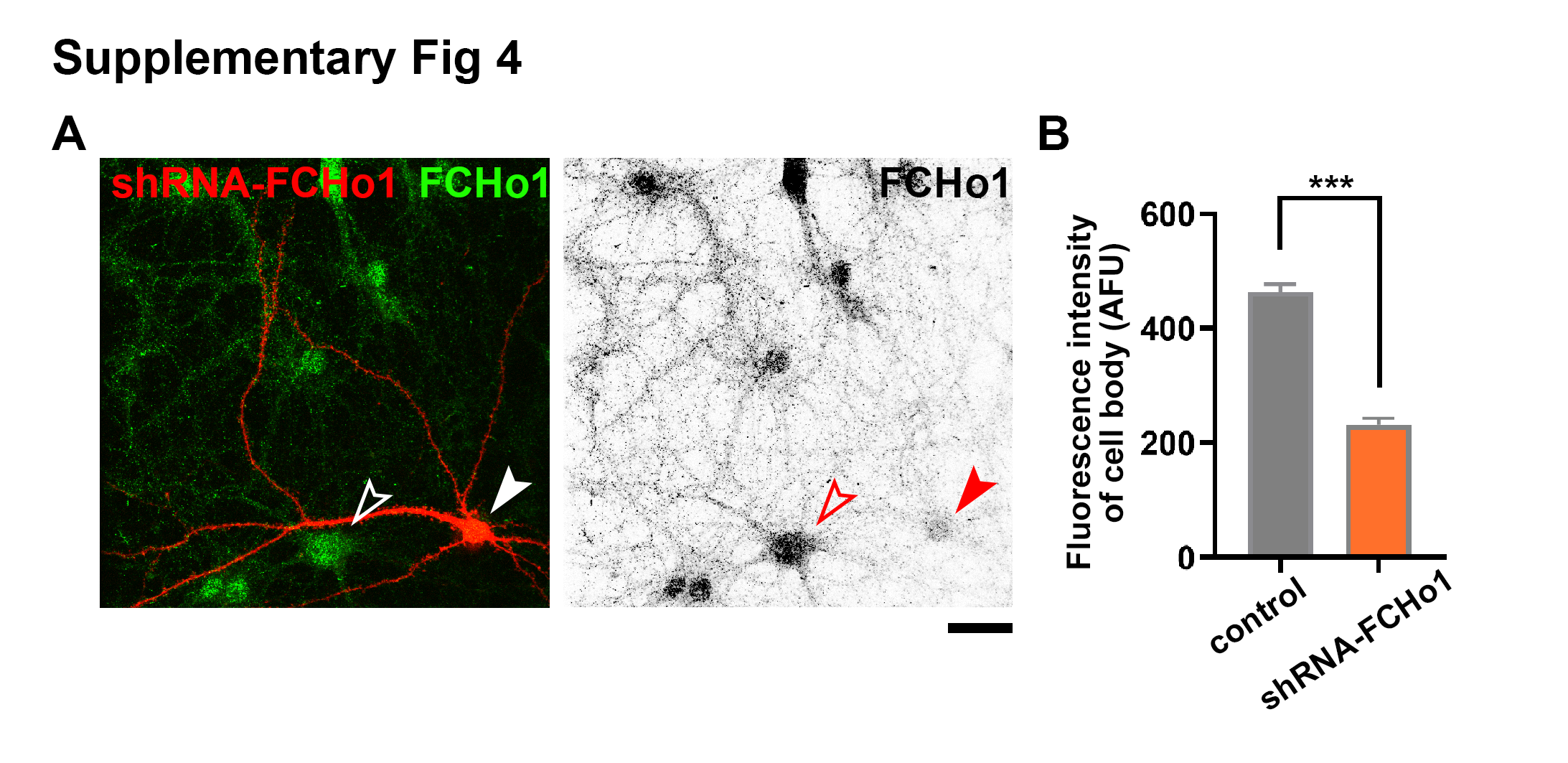

Supplement: Supplementary file 2 [file Image4.tif]

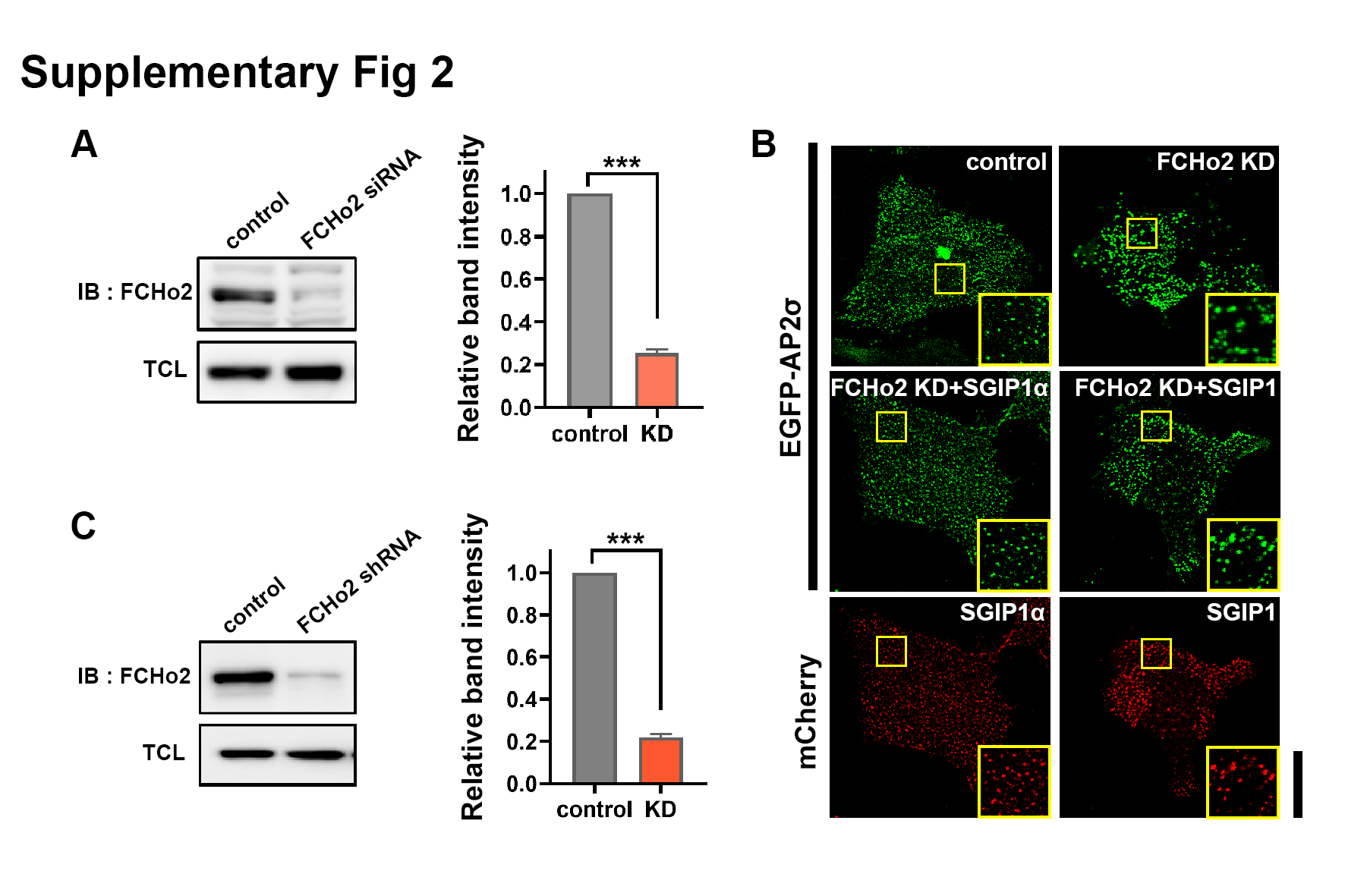

Supplement: Supplementary file 3 [file Image2.tif]

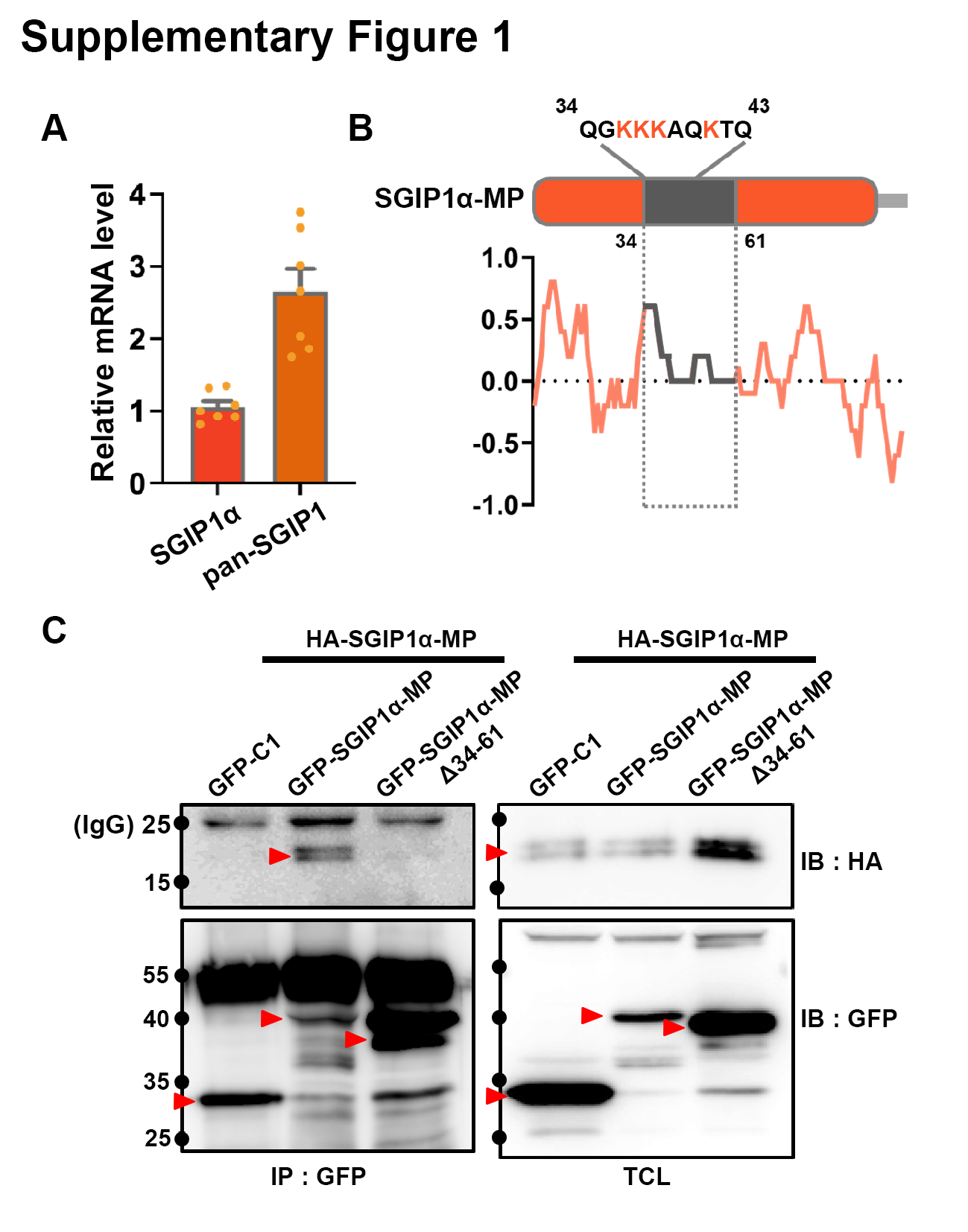

Supplement: Supplementary file 4 [file Image1.tif]
