# Supplementary material for: SGIP1α, but Not SGIP1, is an Ortholog of FCHo Proteins and Functions as an Endocytic Regulator
Source: Front Cell Dev Biol. 2021 Dec 24;9:801420. doi: 10.3389/fcell.2021.801420 (PMC8740024; doi:10.3389/fcell.2021.801420)
Supplement: Supplementary file 5 [file DataSheet1.docx]

**Supplementary Table 1. List of different SGIP1 isoforms used in previous studies.**

| **Article** | **Species** | **Length** | **Additional region in MP** |
| --- | --- | --- | --- |
| Trevaskis *et al*, 2005 ^#1^ | *Psammomys obesus*  Human | 827  828 | X  X |
| Uezu *et al*, 2007 ^#6^ | Mouse | 854 | O |
| Reider *et al*, 2009 ^#15^ | Mouse  Human | 806  859 | X  O |
| Stimpson *et al*, 2009 ^#9^ | Mouse | 806 | X |
| Dergai *et al*, 2010 ^#4^ | Human | 828 | X |
| Henne *et al*, 2010 ^#7^ | Mouse | 806 | X |
| Li *et al*, 2011 ^#10^ | Mouse | 826 | X |
|  | Mouse | 854 | O |
| Uezu *et al*, 2011 ^#8^ | Mouse | 854 | O |
| Cummings *et al*, 2012 ^#2^ | *Psammomys obesus* | 827 | X |
| Hollopeter *et al*, 2014 ^#5^ | Mouse | 854 | O |
| Umasankar *et al*, 2014 ^#16^ | Human | 854 | O |
|  | Mouse | 806 | X |
| Hajkova *et al*, 2016 ^#13^ | Mouse | 806 | X |
| Ma *et al*, 2016 ^#14^ | Mouse | 806 | X |
| Shimada *et al*, 2016 ^#18^ | Human | 828 | X |
| Petko *et al*, 2018 ^#17^ | Human | 828 | X |
| Zhang *et al*, 2018 ^#19^ | Human | 828 | X |
| Lee *et al*, 2019 ^#24^ | Mouse | 854 | O |
| Dvorakova *et al*, 2021 ^#11^ | Mouse | 854 | O |
| Mishra *et al*, 2021 ^#12^ | Mouse | 854 | O |

**#number indicates the reference number.**

**Supplementary Table 2. List of siRNA and shRNAs sequences used in this study.**

| **si/shRNA** | **Targeting Species** | **sequence** |
| --- | --- | --- |
| Human FCHO2 siRNA  (M-024508-01-0005, siGENOME SMARTpools, Dharmacon) | Human | UAUGAAACAUGGACAGAUA  CUACCAUAGAGGAGGCAUA  AGGAGUAGAUUUCGAACUU  GGGUUAUAGUGUAAUAUCA  (A mixture of 4 siRNAs) |
| FCHo1 shRNA | Rat  (CDS 97-115) | GATGATGAGGAGCCTAGA |
| FCHo2 shRNA | Human  (CDS 178-198) | GCAAGCAATTATTCACAACTT |

**Supplementary Figure Legends**

**Supplementary Figure 1. SGIP1/SGIP1α RT-PCR, a charge distribution plot of SGIP1α-MP domain and homo-oligomerization assay.** (A) RT-qPCR quantification of relative mRNA levels of pan-SGIP1 and SGIP1α in mouse hippocampal neurons. The bars represent the mean from 7 samples each analyzed in triplicate PCR runs. (B) A charge distribution plot of the mouse SGIP1α-MP domain. The gray area indicates the additional region (34-61 a.a). (C) Co-immunoprecipitation assay to confirm the homo-di/oligmerization of HA-SGIP1α-MP with EGFP-SGIP1α-MP, but not with EGFP or EGFP-SGIP1α-MP∆34-61. HEK293T cell lysates co-transfected with HA-SGIP1α-MP and EGFP-SGIP1α-MP, EGFP or EGFP-SGIP1α-MP∆34-61 were immunoprecipitated with anti-GFP antibody followed by immunoblotting with anti-HA or anti-GFP antibody. Red arrowhead indicated as follows: HA-SGIP1α-MP (~20 KDa) in IB:HA panel; EGFP (~28 KDa), EGFP-SGIP1α-MP (~40 KDa), and EGFP-SGIP1α-MP∆34-61 (~ 37 KDa), respectively in IB:GFP panel. IgG: Immunoglobulin-G light chain, IP: immunoprecipitation, IB: Immunoblotting. TCL: Total cell lysates

**Supplementary Figure 2. SGIP1α, not SGIP1, reverses the altered AP2 sigma subunit patterning in FCHo2 KD cells.**

(A, C) HeLa cells were transfected with siRNA-FCHo2 (A) or shRNA-FCHo2 (C), and the KD efficiency by each KD was confirmed by Western blotting with an anti-FCHo2 antibody. ***p < 0.001. Error bars indicate mean ± s.e.m. (B) Representative images of EGFP-tagged AP2 sigma subunit (σ) in HeLa cells. HeLa cells transfected with siRNA-human FCHo2 showed enlarged and irregularly distributed patterning of the AP2σ where co-expression of mouse SGIP1α, not SGIP1, restored regular distribution. Insets: magnified images of the region enclosed by yellow rectangles in the low magnification images. Scale bars: 20 μm.

**Supplementary Figure 3. SGIP1α 4KQ and Δ34-61 failed to rescue the endocytic defects caused by FCHo depletion.** (A) Representative fluorescence images of transferrin uptake in HeLa cells. Yellow dotted lines indicate cells transfected with shRNA-human FCHo2. Scale bar: 20 μm. (B) A plot of relative fluorescence signals of transferrin-Alexa647 measured at 5 min of transferrin treatment in HeLa cells in (A). One-way ANOVA followed by Tukey’s HSD test. n = 3 (15 cells per construct for each independent experiment). ***p < 0.001. Error bars indicate mean ± s.e.m.

**Supplementary Figure 4. FCHo1 shRNA efficiently depletes FCHo1 in hippocampal neurons.** (**A**) Primary rat hippocampal neurons transfected with or without shRNA targeting FCHo1 were stained with anti-FCHo1 antibody followed by Alexa 488-conjugated secondary antibody. A filled arrowhead indicates the cell bodies of the shRNA-transfected neuron and a blank arrowhead indicates the control neuron. Expression levels of FCHo1 were measured at cell bodies to avoid possible spatial overlap with other cells. Scale bar: 20 μm. (**B**) Quantification of FCHo1 expression levels in the cell body. N = 10. ***p < 0.001. Student’s *t*-test; Error bars indicate mean ± s.e.m.

**Supplementary methods**

Animal experiments were approved by the Institute of Animal Care and Use Committee (IACUC, Approval ID number: SNU-100930-5) of Seoul National University, Korea. All experiments were carried out in accordance with approved guidelines and regulations.

**DNA constructs and antibody**

Full-length mouse SGIP1α (NM_001285852.1) was generated from GFP-SGIP1, kindly provided by Marek Michalak (University of Alberta, Edmonton, Alberta, Canada), and cloned into mCherry-C1 or FLAG-C1 vector. By PCR site-directed mutagenesis, we prepared several mouse SGIP1 mutation constructs: GFP/HA-SGIP1α-MP (aa 1-115), GFP-SGIP1α-ΔMP (aa 98-854), GFP-SGIP1α-MPΔ34-61 (aa 1-33, 62-115), GFP-SGIP1α-MP 4KQ (aa 1-115, K36Q/K37Q/K38Q/K41Q). FCHo1 targeted small hairpin RNA (shRNA) was designed from the rat FCHo1 sequence (NM_001395712) targeting 97-115 bp and cloned into the pSiren-U6-mRFP vector (Clontech, Palo Alto, CA). FCHo2 targeted shRNA was designed from the human FCHo2 sequence (NM_138782.3) targeting 178-198 bp and cloned as described above.

Small-interfering RNA (siRNA) against human FCHo2 was from Dharmacon (M-024508-01-0005, siGENOME SMARTpools, Lafayette, CO). GFP-CLC was kindly provided by Pietro De Camilli (Yale University, New Haven, CT). Synaptophysin1-pHluorin was provided by Dr. Leon Lagnado (Medical Research Council). All constructs were verified by sequencing. The following antibodies were used: Anti-GFP rabbit polyclonal antibody was custom made by affinity-purification; rabbit anti-Tubulin-β (Abcam, Cambridge, England); rabbit anti-SGIP1 (Abcam, ab102839); rabbit anti-HA (Covance, Princeton, NJ); rabbit anti-FCHo1 (ThermoFisher); rabbit anti-FCHo2 (Abcam); mouse anti-FLAG (Sigma, St.Louis, MO, F3165); HRP-conjugated anti-rabbit IgG secondary antibody (Jackson ImmunoResearch, WestGrove, PA).

**Primary Neuron Culture and Transfection**

Primary rat hippocampal neurons derived from embryonic day 18 Sprague Dawley fetal rats of either sex were prepared as described previously ^28^. Briefly, hippocampi were dissected, dissociated with papain, and triturated with a polished half-bore pasteur pipette. 2.5 x 10^5^ cells were resuspended in Hank’s Balanced Salt Solution (HBSS; HyClone, Logan, UT) supplemented with 0.6 % glucose, 1 mM pyruvate, 2 mM L-glutamine, and 10 % (v/v) FBS (HyClone) and plated on Poly-D-lysine-coated glass coverslips in a 60-mm Petri dish. 4 hr after plating, the medium was replaced with neurobasal medium (Invitrogen) supplemented with 2 % (v/v) NS21, 0.5 mM L-glutamine. 4 mM 1-β-D-cytosine-arabinofuranoside (Ara-C; Sigma) was added as needed. Neurons were triply transfected with shRNA-FCHo1, synaptophysin1-pHluorin, and either mouse SGIP1α or SGIP1 using a modified calcium-phosphate method. Briefly, 6 μg of DNA and 9.3 μl of 2 M CaCl_2_ were mixed in distilled water to a total volume of 75 μl and the same volume of 2x BBS [50 mM BES, 280 mM NaCl, and 1.5 mM Na_2_HPO_4_ (pH 7.1)] was added. The cell culture medium was completely replaced by transfection medium (MEM; 1 mM sodium pyruvate, 0.6 % glucose, 10 mM HEPES, 1 mM Kynurenic acid, and 10 mM MgCl_2_, pH 7.71), and the DNA mixture was added to the cells and incubated in a 5 % CO_2_ incubator for 60 minutes (min). Cells were washed with a washing medium, pH 7.30, and then returned to the original culture medium. Neurons were triply-transfected at a ratio of 1:1:1 at DIV 8-9 and analyzed at DIV 16-21.

**RT-qPCR**

Mouse hippocampal cDNA was prepared from 150 ng/ml of total RNA using a Maxime RT PreMix kit (iNtRON Biotechnology, Seoul, South Korea). Then, real-time quantitative PCR (qPCR) was performed using SYBR Green Fast mix (Applied Biosystems, Waltham, MA) and the following primer pairs: mouse SGIP1α, 5’- CCA GAA GAC TCA GCT CCT CC-3’ (forward) and 5’- CTG CTG AGA CAG GGT TAA GGA-3’ (reverse); pan-mouse SGIP1, 5’- AAG TCG CTG AGC AGA CCT TC-3’ (forward) and 5’- CTC TCT GTC CCA ACC CAC AC-3’ (reverse); and mouse Rps18, 5’- ACT TTT GGG GCC TTC GTG TC-3’ (forward) and 5’- GCC CAA AGA CTC ATT TCT TCT TG-3’ (reverse). Expression levels of target mRNAs were analyzed using the DDCt method and were normalized to expression levels of Rps18, a housekeeping gene used as an endogenous control. All fold changes are expressed relative to the control group.

**Homo-oligomerization assay**

To test whether SGIP1α-MP can form homo-di/oligomer, we conducted co-immunoprecipitation assay. HEK293T cells were co-transfected with EGFP-C1, EGFP-SGIP1α-MP, and EGFP-SGIP1α-MPΔ34-61 together with HA-SGIP1-MPα respectively using PEI (Polysciences), and incubated for 48 hr. Cell lysates were prepared in 1 % Triton X-100 buffer (20 mM Tris-HCl, pH 8.0. 137 mM NaCl, 10 % glycerol, 1 % TritonX-100, 2 mM EDTA) containing a protease inhibitor cocktail (Roche, Mannheim, Germany). Then, the samples were clarified by centrifugation 13,200 x g for 20 min, and concentrations were measured with a Bicinchoninic acid (BCA) Protein Assay Reagent kit (Bio-Rad, Hercules, CA). Cell lysates containing 1 mg of total protein were incubated with rabbit anti-GFP (1:1000) antibody for 2 hr at 4 ℃ and then with Protein A-Sepharose beads (GE Healthcare, Uppsala, Sweden) for 2 hr. After extensive washing in lysis buffer and centrifugation, a total of 30 μg proteins from each sample was analyzed by SDS-PAGE (6–12 %), and transferred onto a PVDF (Bio-Rad) membrane which was previously activated in 100 % methanol. The membrane was blocked with 5 % skim milk in TBS-T (10 mM Tris-Cl, pH 7.6, 100 mM NaCl and 0.1 % Tween 20) for 30 min, washed for 5 min, and incubated with rabbit anti-GFP (1:1000) or rabbit anti-HA (1:1000) primary antibody overnight at 4 ℃. The membrane was extensively washed with TBS-T and probed with HRP-conjugated IgG secondary antibody (Jackson ImmunoResearch). Proteins were detected with ECL Western Blotting detection reagents (GE Healthcare) using ImageQuant LAS 4000 (GE Healthcare).

**Protein purification**

All sequence-verified plasmids and 6xHis vector were transformed into BL21 (DE3) *Escherichia coli* cells and the cells grew in 2xYT media containing kanamycin (40 ug/ml) to the turbidity of A600 = 0.6~0.8. Protein expression was induced in cells by incubating the cells for 4 hr with 0.5 mM Isopropyl-B-D-thiogalactopyranoside (IPTG) at 37 °C. Cells were lysed using lysis buffer (25 mM HEPES [pH 7.4], 400 mM KCl, 10 % glycerol, 20 mM imidazole, 0.5 % Triton X-100, 1 mg/ml lysozyme, 0.1 mg/ml DNase1, 1 mM PMSF, protease inhibitor cocktail including 104 μM AEBSF, 80 nM Aprotinin, 4 μM Bestatin, 1.4 μM E-64, 2 μM Leupeptin and 1.5 μM Pepstatin A). Proteins were purified from lysed cell extracts by incubating with Ni-NTA resin for 2 hr 30 min, followed by 3 times washing with buffer containing 25 mM HEPES [pH 7.4], 400 mM KCl, 20 mM imidazole, 10 % glycerol. Proteins were eluted by incubating with elution buffer (25 mM HEPES [pH7.4], 400 mM KCl, 300 mM imidazole) for 20 min. All collected proteins except 6xHis were concentrated using Amicon Centrifugal Filters Units (Merck Millipore, Burlington MA) for 1 hr. Eluted proteins were quantified with Coomassie staining after SDS-PAGE. All experiments after protein induction were performed at 4 °C or on ice.

**GUV preparation and imaging**

Polyvinyl alcohol (PVA) was dissolved in distilled water at 5 % (w/w) concentration by stirring on a hot plate at 90 °C. The dissolved PVA solution was stored at 4 °C and was completely thawed in an oven at 50 °C before use. An aliquot of 200 μl of 5 % PVA was spread on a plasma cleaned 25 mm glass slide, followed by spinning the slide on the mini centrifuge to form a uniform thin PVA layer. The PVA-coated slide was dried at ~50 °C 1 hr in a dry oven. A lipid mixture of 81 mol% POPC, 0.5 mol% DOPE, 15 mol% DOPS, 2.5 mol% DOGS-NTA Ni, and 1 mol% DiI was dissolved in chloroform at 1 mg/ml concentration in a glass vial. An aliquot of 5 μl of the lipid mixture was spread on the dried PVA layer using a Hamilton syringe. Chloroform was evaporated immediately after spreading, the dried glass slide was placed in a vacuum for at least another 1 hr to remove residual chloroform. An O-ring was glued onto the glass slide, followed by injecting 300 μl of GUV swelling buffer to the inside of the O-ring. For swelling, the glass slide was incubated 1 hr 30 min at room temperature in the dark. The GUVs solution inside the O-ring was withdrawn into an E-tube using a pipette with a cut tip and kept at room temperature. For the ideal condition, GUVs were used within 2 days. GUVs were mixed with each His-tagged SGIP1 protein and then imaged using an Olympus IX71 fluorescence microscope, a 60X 1.35 N.A. oil-immersion lens and an Andor Zyla-5.5-CL3 sCMOS camera (Andor Technologies) driven by MetaMorph imaging software (Molecular Devices).

**Transferrin uptake assay**

HeLa cells were starved for 3-4 hr in serum-free DMEM 24 hr after transfection and incubated in serum-free DMEM/HEPES containing 20 mg/ml Transferrin-647 (Molecular Probe, Eugene, OR) for 15 min on ice. Cells were washed with PBS and then incubated at 37 °C in serum-free DMEM/HEPES for 5 min. Then, cells were washed in an acid stripping solution (NaCl 0.5 M and acetic acid 0.2 M, pH 4.5) for 1 min and fixed in 4 % paraformaldehyde. Cells were mounted on a slide glass and then imaged with a 488 or 561 nm laser using a spinning disk confocal microscope (Nikon) as described above. Transferrin-647 intensities were averaged over individual cells and analyzed using ImageJ.

**pHluorin endocytosis assay and image analysis**

Coverslips were mounted in a perfusion/stimulation chamber equipped with platinum-iridium field stimulus electrodes (Chamlide; LCI, Seoul, South Korea) on the stage of an Olympus IX-71 inverted microscope. Primary rat hippocampal neurons were continuously perfused at 34 °C with Tyrode’s solution with the temperature of the chamber and lens controlled by a heating controller system (LCI). 10 μM 6-cyano-7-nitroquinoxaline-2,3-dione (CNQX) and 50 μM of DL-2-amino-5-phosphonovaleric acid (AP-V) were added to the imaging buffer to reduce spontaneous activity and to prevent recurrent excitation during stimulation. Time-lapse images were acquired every 5 s for 5 min using a back-illuminated Andor iXon 897 EMCCD camera driven by MetaMorph Imaging software. From the fifth frame, the cells were stimulated (1 ms, 20–50 V, bipolar) using an A310 Accupulser current stimulator (World Precision Instruments, Sarasota, FL). Quantitative measurements of the fluorescence intensity at individual boutons were obtained by averaging the pixel intensities of the selected area using ImageJ. Net fluorescence changes, for individual boutons, were obtained by subtracting the average intensity of the first four frames (F_0_) from the intensity of each frame (F_t_). They were then normalized to the maximum fluorescence intensity (F_max_−F_0_) and averaged. All fitting was done using an exponential decay equation model in GraphPad Prism 9.0 (GraphPad Software, San Diego, CA). To obtain the endocytic time constant after stimulation, the decay of pH-probe after stimulation was fitted with a single exponential function. Data were collected from ~ 100 boutons of 8-10 neurons in each coverslip and “n” stands for the number of coverslips. Statistical analysis was performed with GraphPad Prism 9.0 (GraphPad Software). Once the normality assumption was satisfied, groups were compared by ordinary ANOVA-test.
